# Hybrid Polyelectrolyte Nanocomplexes for Non-Viral Gene Delivery with Favorable Efficacy and Safety Profile

**DOI:** 10.3390/pharmaceutics14071310

**Published:** 2022-06-21

**Authors:** Gabriele Maiorano, Clara Guido, Annamaria Russo, Andrea Giglio, Loris Rizzello, Mariangela Testini, Barbara Cortese, Stefania D’Amone, Giuseppe Gigli, Ilaria Elena Palamà

**Affiliations:** 1 Nanotechnology Institute of National Research Council, CNR-NANOTEC, Monteroni Street, 73100 Lecce, Italy; gabriele.maiorano@nanotec.cnr.it (G.M.); clara.guido@nanotec.cnr.it (C.G.); annamaria.russo@nanotec.cnr.it (A.R.); andrea.giglio@nanotec.cnr.it (A.G.); mariangela.testini@nanotec.cnr.it (M.T.); stefania.damone@nanotec.cnr.it (S.D.); giuseppe.gigli@unisalento.it (G.G.); 2 Department of Mathematics and Physics, University of Salento, Monteroni Street, 73100 Lecce, Italy; 3 Department of Pharmaceutical Sciences (DISFARM), University of Milan, G. Balzaretti 9 Street, 20133 Milan, Italy; loris.rizzello@unimi.it; 4 National Institute of Molecular Genetics (INGM), Francesco Sforza 35 Street, 20122 Milan, Italy; 5 Nanotechnology Institute of National Research Council, CNR-NANOTEC, c/o La Sapienza University, Piazzale Aldo Moro, 00185 Rome, Italy; barbara.cortese@nanotec.cnr.it

**Keywords:** hybrid nanovector, non-viral gene delivery, safety profile

## Abstract

The development of nanovectors for precise gene therapy is increasingly focusing on avoiding uncontrolled inflammation while still being able to effectively act on the target sites. Herein, we explore the use of non-viral hybrid polyelectrolyte nanocomplexes (hPECs) for gene delivery, which display good transfection efficacy coupled with non-inflammatory properties. Monodisperse hPECs were produced through a layer-by-layer self-assembling of biocompatible and biodegradable polymers. The resulting nanocomplexes had an inner core characterized by an EGFP-encoding plasmid DNA (pDNA) complexed with linear polyethyleneimine or protamine (PEI or PRM) stabilized with lecithin and poly(vinyl alcohol) (PVA) and an outer layer consisting of medium-molecular-weight chitosan (CH) combined with tripolyphosphate (TPP). PEI- and PRM-hPECs were able to efficiently protect the genetic cargo from nucleases and to perform a stimuli-responsive release of pDNA overtime, thus guaranteeing optimal transfection efficiency. Importantly, hPECs revealed a highly cytocompatible and a non-inflammatory profile in vitro. These results were further supported by evidence of the weak and unspecific interactions of serum proteins with both hPECs, thus confirming the antifouling properties of their outer shell. Therefore, these hPECs represent promising candidates for the development of effective, safe nanotools for gene delivery.

## 1. Introduction

Genetic therapies are based on the successful delivery of nucleic acids towards target cells, with the aim to selectively modulate the expression of specific genes. At the moment, three main categories of nucleic acid delivery have been developed: (i) direct delivery, (ii) viral delivery, and (iii) non-viral delivery [1]. The direct administration of genetic material limits its therapeutic efficacy due to several factors such as the action of serum nucleases, rapid renal clearance, uptake by macrophages, and off-target effects [2]. On the other hand, the viral delivery of nucleic acids guarantees high transfection efficacy for the stable insertion in the genome of the host cell [3]. As a matter of fact, their application in CAR-T therapy has proven their effectiveness [4], allowing the gene encoding for the chimeric antigen receptor (CAR) to be transferred into T lymphocytes. Despite the successful therapeutic results achieved with viral vectors, their use is often hampered by several disadvantages, such as a limited capacity for the allocable transgenes, being difficult to handle, potential insertional mutagenesis, safety issues, and excessive production costs [5]. Additionally, viral vectors might induce inflammatory responses, leading to the production of cytokines and chemokines. Cytokine bursts can gather neutrophils, macrophages, and natural killer cells to the site of infection where, at the same time, an adaptive immune response can be triggered through antigen-presenting cell (APC) activation [6]. To overcome these disadvantages, several efforts have addressed the development of alternative strategies for genetic therapy in the last decade. The first strategies were based on the injection of naked DNA through electroporation. The “gene guns” consisted of DNA bound to gold nanoparticles that were delivered to the cells, together with chemical strategies employing cationic liposomes (lipoplexes) or polymers (polyplexes) to prevent the degradation of the nucleic acids [7]. Nevertheless, while viruses have evolved sophisticated mechanisms to overcome the biological barriers, the non-viral nanocarriers must face several hurdles before efficiently delivering their payload. The major challenge is represented by the crossing of the cell membrane, followed by the cargo delivery within the appropriate subcellular compartment [8]. In this frame, stimuli-responsive polymers, i.e., macromolecules able to deliver their cargo upon specific changes in the surrounding physiological conditions, represent a promising opportunity for an effective viral vector replacement [9]. However, there is a general need to increase the nanovector transfection efficacy [10] while ensuring biocompatibility and non-inflammatory properties.

To address this challenge, non-viral hybrid polyelectrolyte nanocomplexes (hPECs), based on stimuli-responsive polymers such as protamine (PRM), linear polyethyleneimine (PEI), and chitosan (CH), are proposed here as a tool for the production of non-inflammatory, highly effective transfection agents with important implications in cancer immunotherapy for CAR-T production [11]. The first two polymers were separately employed to condense nucleic acids by the neutralization of their negative charge in order to confer stability and protection against nucleases. Additionally, PRM-based polyplexes enhance the efficiency of gene transfections [12,13,14] and could orient DNA to the nuclei of cells [15]. In the same way, linear PEI was exploited for the ability to strongly interact with nucleic acids in a whole range of PEI/nucleic acid ratios [16], thus producing stable polyplexes in physiological fluids with high diffusibility [17] that could enter nuclei [18]. Chitosan was employed to cover hPECs, as it is a biodegradable and biocompatible cationic polysaccharide exhibiting bioadhesive and penetration-enhancing properties [19]. Furthermore, the deacylation degree as well as the molecular weight of chitosan were shown to differently affect the immune response against chitosan-based nanomaterials [20]. In particular, chitosan with a molecular weight above 156,000 was shown to inhibit NF-κB activation without triggering any inflammatory process [21]. This led us to employ medium-molecular-weight chitosan to build hPECs in order to avoid NF-κB translocation in the nucleus. To demonstrate the potential therapeutic applications of the proposed hPECs together with their non-inflammatory framework, human monocyte-derived macrophages, MΦ (THP-1) in different polarization states (i.e., M0, M1, and M2) were observed [22]. Finally, by monitoring the production and localization of NF-κB, MΦ were demonstrated to react differently to the nanoparticles as a function of their polarization states [23].

## 2. Materials and Methods

### 2.1. Materials

All chemicals were of analytical grade and were used as received from Sigma-Aldrich, (Merck KGaA, Darmstadt, Germany)without any further purification. Cell culture media were also obtained from Sigma-Aldrich (Merck KGaA, Darmstadt, Germany). SYBR-safe was purchased from Thermo Fisher (Waltham, MA, USA). Coomassie brilliant blue (R-250) was obtained from BioRad (Milan, Italy). EGFP plasmid (4.7 kb) encoding the enhanced green fluorescence protein (EGFP) was obtained by Novoprolabs (cod. V012024). Human blood was obtained from informed healthy donors under guidelines (prot. CE n. 68 of 26 November 2021) approved by the Ethics Committee of ASL Lecce (Italy). All healthy donators gave their informed consent in accordance with the Declaration of Helsinki.

### 2.2. Purification of Medium-Molecular-Weight (MMW) Chitosan

Briefly, 4 g of MMW chitosan (190,000–310,000 Da, 75–85% deacetylated) was dissolved in 20 mL of 1 M NaOH by stirring for 12 h at a temperature of 70 °C. Then, the suspension was cooled down, and the precipitate was recovered by filtration. The obtained white slurry was subsequently dissolved in 200 mL of 0.1 M acetic acid solution and filtered again to remove acid-insoluble impurities. Next, the pH was increased to a value of 8 by adding the appropriate amount of NaOH, and the precipitates were collected, washed three times with ultrapure water, and finally freeze-dried.

### 2.3. Preparation of hPECs

**Figure 1:**
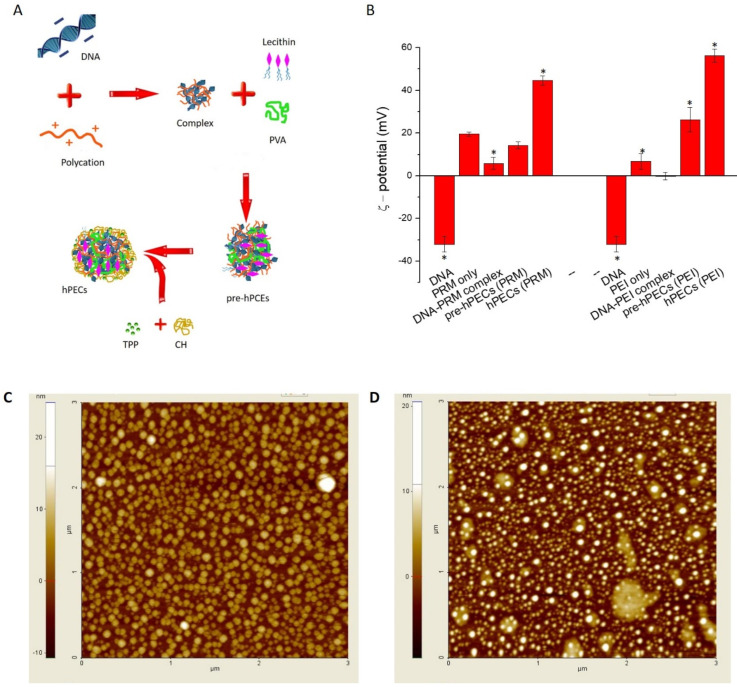
Schematic illustration of the synthetic procedure (**A**). In panel (**B**), mean zeta potential values of protamine-based and linear polyethyleneimine-based hPECs at each step of preparation. DNA refer to EGFP plasmids. Representative AFM images of dried PRM-hPECs (**C**) and dried PEI-hPECs (**D**). Scale bars, 1 μm. Representative measurements of three distinct sets of data, * indicates *p*-values of <0.05 for Student’s *t*-test.

Polyethylenimine (PEI, linear, average MW (Mn) 10,000, PDI ≤ 1.3, data from the supplier) was dissolved in ultrapure water at a concentration of 50 mg/mL. Protamine sulfate (PRM, MW 5000–10,000) was dissolved in 0.1 M NaCl at a concentration of 50 mg/mL. Poly(vinyl alcohol) (PVA, average MW 13,000–23,000, 98% hydrolyzed) was suspended in ultrapure water at a concentration of 1 mg/mL by heating the solution at about 70 °C while stirring until complete dissolution. L-α-Lecithin from soybean (94% phosphatidylcholine and less than 2% triglycerides) was dissolved in ethanol at a concentration of 30 mg/mL. Purified MMW chitosan was dissolved in 1% HCl at 4 mg/mL, and sodium tripolyphosphate (TPP) was dissolved in ultrapure water at 20 mg/mL. Prior to use, each component was then individually filtered with 0.45 µm PVDF syringe filters. First of all, pre-complexes between the plasmid DNA and PEI or PRM were prepared by mixing and stirring plasmid DNA into the polymer solution at a DNA/PRM or DNA/PEI ratio of 1:100 (*w*/*w*) at a final DNA concentration of 100 ng/µL. Stirring was continued for 30 min at room temperature. Then, the appropriate quantities of pre-complexes were added to 1 mL of PVA solution under vigorous stirring. Immediately after, lecithin solution was poured inside the solution that was left stirring for 30 min. In the meantime, in a separated vial, TPP solution was added into 2.5 mL of chitosan, and the mixture was stirred for 10 min and then added to the main solution to obtain a nanoformulation with a final DNA concentration of 10 ng/µL. The final formulations (DNA/PEI- or DNA/PRM-based hPECs) were left stirring for 30 min at room temperature, then the dispersions of hPECs were extensively dialyzed against water by employing Spectra/Por^®^ 6 dialysis tubing, MWCO 3500 Da following the manufacturer’s protocol. Empty hPECs were prepared by replacing the plasmid DNA with the appropriate amount of an aqueous solution of heparin sodium salt at 100 μg/mL and then following the same reaction scheme (see Figure 1A) already described for the production of DNA/PEI- or DNA/PRM-based hPECs. Fluorescent hPECs were prepared with the same procedure by employing PRM or PEI labeled with fluorescein isothiocyanate (FITC), as reported by Saito and Saitoh, in order to obtain fluorescent labeling with minimal neutralization of the positive charges present in PRM or PEI [24]. Briefly, a stock solution of FITC in dimethyl sulfoxide (DMSO) was added to 20 mg/mL PEI or 30 mg/mL PRM in phosphate-buffered saline (PBS) at a final concentration of 0.10 mM. After 3 h of incubation, dialysis was performed as already described to remove unbound FITC. The resulting FITC-PRM and FITC-PEI were stored at −80 °C and then lyophilized to be used for the self-assembly of fluorescent hPECs. 

### 2.4. Particle Size and Zeta Potential Analyses

The mean hydrodynamic diameter and zeta potential (ζ) values were measured by employing a dynamic light-scattering (DLS) Zetasizer Nano-ZS90 (Malvern Instruments, Malvern, UK), equipped with a 4.0 mV He-Ne 633 nm laser. The measurements of zeta potential were carried out in each step of the assembly of hPECs in order to identify the optimal concentrations of each component leading to monodisperse and stable nanovectors. The measurements of hydrodynamic diameter were carried out by dispersing dialyzed dispersions of hPECs in pH 7.4 PBS.

### 2.5. Atomic Force Microscopy (AFM)

Samples were prepared by applying a drop of the hPEC suspension to SiO_2_ wafers that were then dried overnight. The analysis was performed in non-contact mode with a Park Systems XE-100 AFM with commercially available AFM tips (Nanosensors PPPNCHR) with a nominal force constant of 42 N/m. The particle size distribution analyses were performed by drawing a linear region of interest to extract measures of at least 100 particles for each image. A Gaussian fit was interpolated on the frequency distribution obtained.

### 2.6. Gel Retardation Assay

The electrophoretic mobility of the pre-complexes and hPECs was determined using 1% agarose gel in TBE 1X (Tris/Borate/EDTA) buffer with 2 µL of SYBR-safe at 90 V for 30 min. The gel was visualized under a UV transilluminator (Chemidoc, Bio-Rad, Milan, Italy).

### 2.7. Release of Encapsulated DNA from hPECs

A known amount of hPECs were suspended in pH 7.4 PBS 1X, and the free DNA was isolated by ultracentrifugation at 50,000× *g* for 15 min at 15 °C using a Optima MAX-XP (Beckman Coulter, Brea, CA, USA) and then quantified with a Nanodrop ND-ONEC-W (ThermoFisher, Waltham, MA, USA). The amount of free DNA was then used to estimate the encapsulation efficiency and was employed in the calculations of DNA release under different conditions. The release of the encapsulated DNA from hPECs over time was performed at two different physiological conditions: pH 4.5, which is the lysosomal pH, and pH 7.4, which is the physiological pH value of living cells (cytosol). Briefly, hPECs were suspended in 10 mL of pH 4.5 and pH 7.4 PBS and maintained at 37 °C under stirring. At specified time intervals, aliquots of hPECs were centrifuged at 50,000× *g* for 15 min at 15 °C. The absorbance of the supernatant at 260 nm was then measured in order to quantify the release of plasmid DNA. Additionally, hPECs prepared with PRM were suspended in pH 7.4 PBS containing 0.5 mg/mL proteinase K, and the DNA released over time was assessed as described above; similarly, PEI-hPECs were incubated with 0.1 mg/mL heparin in pH 4.5 PBS to evaluate the DNA displacement induced by competitive binding [25,26].

### 2.8. DNAse and Serum Protection Assay

The ability of hPECs to complex and protect pDNA from DNase and nucleases was analyzed by incubating DNA-loaded hPECs with DNase I (1U) or 10% fetal bovine serum (FBS) with constant stirring (600 rpm) at 37 °C. The incubations were allowed to proceed for variable time periods of 30, 90, and 180 min and then stopped with 1% SDS. To measure the intactness of the pDNA displaced from the nanocomplexes, the samples were then electrophoresed on 1.2% agarose gel in 1X TAE for 1 h at 90 V. As a positive control, plasmid DNA was used. Bands were visualized with SYBR-safe staining under a UV transilluminator (Chemidoc, Bio-Rad, Milan, Italy).

### 2.9. Protein Corona Analysis by Means of SDS-PAGE

The protein corona was analyzed by SDS-PAGE. First, PEI- and PRM-hPECs as well as empty PEI- and PRM-hPECs were incubated in RPMI full supplemented cell culture medium (10% FBS) for 1 h, 6 h, and 24 h at 37 °C under gentle shaking and were subsequently subjected to centrifugation at 50,000× *g* for 15 min at 4 °C. The obtained pellets were washed two times with sterile 1X PBS (pH 7.4) by repetitive centrifugal processes in order to separate the unbound serum proteins from the hPECs. The obtained pellets were resuspended in a small volume of 1X PBS and then protein quantification and separation were performed. Protein quantification was performed by a Bradford assay following the manufacturer’s protocol. The quantity of proteins was assessed by employing a calibration curve based on bovine serum albumin (BSA) as a protein standard (0.1–1.2 mg/mL). Protein separations were performed by SDS-PAGE; briefly, the quantified protein-hPEC complexes were mixed with 2 × Laemmli buffer (Bio-Rad, Milan, Italy) and 0.2 M dithiothreitol (DTT), then heated to 90 °C and loaded into 15% SDS-PAGE mini-gels. As a marker, prestained Precision Plus Protein Dual Color standards (Bio-Rad, Milan, Italy) were used. The electrophoresis was carried out at a constant voltage of 100 V. The resulting gels were stained with Coomassie brilliant blue G-250 and then treated with a destaining solution composed of an aqueous solution of methanol (40%) and acetic acid (10%). Finally, gels were acquired using the Chemidoc imaging system (Bio-Rad, Milan, Italy).

### 2.10. Cell Culture

Human monocytic cell line (THP-1) was cultured in RPMI medium supplemented with 10% heat-inactivated FBS, 100 U/mL penicillin, 100 mg/mL streptomycin, and 4 mM L-glutamine. The cell culture was maintained in a humidified incubator at 37 °C and 5% CO_2_.

### 2.11. Cellular Uptake and Transfection Study

Cellular uptake was assessed by flow cytometry (BD ACCURI C6) on the THP-1 cell line counting 10,000 ungated cells. Briefly, THP-1 cells were plated into a 24-well plate at a concentration of 10^5^ cells/well. The cells were treated with FITC-conjugated hPECs at a concentration of 50 µg/mL for 24 h. For qualitative uptake analysis, THP-1 cells (10^5^ cells/mL) were treated with the same amount of fluorescent hPECs for 24 h, fixed with 4% paraformaldehyde for 5 min, and analyzed using a Leica TCS SP5 confocal microscope (Leica Microsystem GmbH, Mannheim, Germany). For transfection efficacy, THP-1 cells were plated at a concentration of 10^5^ cells/well. Cells were treated with pEGFP-loaded hPECs at a concentration of 100 ng/well, and the transfection efficiency was assessed on days 5, 10, 15, and 20. As a comparison, Lipofectamine 3000 (ThermoFisher, Waltham, MA, USA) was used to transfect the same pEGFP. The transfection rate was assessed by flow cytometry counting 10,000 ungated cells (CytoFLEX S, Beckman-Coulter, Brea, CA, USA).

### 2.12. THP-1 Polarization

To keep the THP-1 cell line undifferentiated, fresh medium was supplemented with 0.05 mM 2-mercaptoethanol (BME). For M0 polarization, a medium containing BME was replaced with fresh RPMI supplemented with phorbol 12-myristate 13-acetate (PMA) at a final concentration of 5 ng/mL, and cells were incubated for 48 h. For M1 polarization, all the medium was gently removed and replaced overnight with fresh RPMI supplemented with lipopolysaccharides (LPS) from *Escherichia coli* O111:B4 at a final concentration of 20 ng/mL and interferon-gamma (IFN-γ) at a final concentration of 20 ng/mL. For M2 polarization, all the medium was gently removed and replaced with fresh RPMI supplemented with interleukin-4 (IL-4) at a final concentration of 100 ng/mL and interleukin-13 (IL-13) at a final concentration of 100 ng/mL. For M2 polarization, cells were incubated for 4 days.

### 2.13. Nanotoxicity Assessment

MTT metabolic assay. THP-1 cell line unpolarized and polarized M0, M1, and M2 cells were seeded on 96-well plates at a density of 10^4^ cells/well and cultured according to the previously described polarization protocol. After each polarization checkpoint, cells were treated with hPECs for 24 h. An MTT assay was conducted according to the manufacturer’s indication (Sigma-Aldrich), and cell viability was determined by absorbance at 570 nm with the formula:

(1) $$Cell\;viability\;\left(\%\right)=\frac{OD_{sample}}{OD_{control}}\times 100$$

where the control sample refers to the THP-1 cell line in normal conditioning media without any treatment.

Live/Dead assay and cell cycle investigation. Cell apoptosis and DNA distribution in the cell cycle were analyzed by a cytofluorimetric analysis. Unpolarized and polarized M0, M1, and M2 THP-1 cells (10^5^ cells) were treated with different hPECs formulations (50 µg/mL) for 24 h at 37 °C and 5% CO_2_. After incubation, cells were washed with 1X PBS and stained with Annexin V-FITC/PI according to the manufacturer’s instructions (Abcam, Cambridge, UK). The cell apoptosis and cell cycle distribution were determined by analyzing 10,000 ungated cells using a Beckman-Coulter CytoFLEX S. 

Cellular ROS, NO, and SOD detection assay. Cytofluorimetry analyses of cellular ROS and NO productions and SOD activity inhibition were performed by analyzing 10,000 ungated cells in a Beckman-Coulter CytoFLEX S. Briefly, unpolarized and polarized M0, M1, and M2 THP-1 cells (10^5^ cells) were treated with different hPECs formulations (50 µg/mL) for 24 h at 37 °C and 5% CO_2_. After incubation, cells were washed with 1X PBS and stained with ROS, NO, and SOD according to the manufacturer’s instructions (Abcam). 

Inflammatory analysis. THP-1 cells were seeded on top of round slides that fit into a 24-well plate at a concentration of 5 × 10^4^/well and cultured following the polarization protocol. After each polarization checkpoint, the cells were treated with FITC-conjugated empty hPECs or with pEGFP-loaded hPECs for 24 h. After incubation, the cells were fixed with 3.7% formaldehyde and permeabilized with Triton X-100 at 0.2%. The slides were then incubated overnight at +4 °C with an anti-NF-κB primary antibody (rabbit) at a 1:200 dilution. Next, the slides were washed and incubated for 60 min at room temperature with TRITC-conjugated Goat anti-rabbit IgG secondary antibodies. The samples were then analyzed with fluorescent microscopy (EVOS^TM^ M7000, Invitrogen Waltham, MA, USA) or using a Beckman-Coulter CytoFLEX S (10,000 ungated cells). 

Hemolytic analysis. An investigation of the hemolytic effect was carried out by incubating different hPEC formulations and DNA-Lipofectamine 3000 with 2% human blood for 1 h at 37 °C with shaking at 600 rpm. hPECs were removed by centrifugation, the supernatant absorption was evaluated at a wavelength of 540 nm by a UV-visible spectrophotometer (Varian Cary^®^ 300 Scan; Varian Instruments, Palo Alto, CA, USA), and the hemolytic rate was calculated as reported in ref. [27].

### 2.14. Statistical Analysis

Three independent experiments were performed, and the results were expressed as means ± standard deviations. Statistical analyses used Student’s *t*-test or a one-way ANOVA.

## 3. Results and Discussion

### 3.1. hPEC Synthesis and Characterizations

The produced hPECs are based on the proper combination of plasmid DNA, PEI or PRM, PVA, lecithin, and chitosan-TPP. The concentration of each building block was properly adjusted by monitoring the ζ-potential values at each step of the preparation as a key indicator of the colloidal stability and dispersion status. Polyplexes between PRM or linear PEI and EGFP-encoding pDNA showed surface charges quite near to neutrality (Figure 1B). This allows the negatively charged nucleic acid to be fully complexed by the cationic polymers (PRM or PEI), thus ensuring the stability of the formed polyplexes. Next, PVA and lecithin were added to enhance the stability and prevent aggregation. The surface charges of the dispersions shifted to positive values of +14.2 mV for the precomplexes based on PRM and of +26.3 mV for the PEI-based ones. These results highlight the ability of PVA and lecithin to assemble in the inner core of the nanostructures, thus exposing polycations at the surface. The nanocomplexes gained stability thanks to the steric PVA-lecithin interaction, with a consequent prevention of colloidal aggregation/agglomeration. In the final production step, the addition of chitosan-TPP conferred a stronger cationic property to both PRM-hPECs and PEI-hPECs, with surface charge values of +44.7 and +56.3 mV, respectively (Figure 1B and Figure S1B). 

AFM-based morphological investigations of the PRM- and PEI-based hPECs depicted spherical, uniform nanoparticles with a good degree of monodispersion and with the absence of aggregation (Figure 1C,D). The particle size distribution analyses (Supplementary Figure S1C,D) reported diameters of 86.9 ± 9.5 nm and 99.3 ± 14.6 nm for PRM-hPECs and PEI-hPECs in dried conditions, respectively. The dynamic light scattering analyses (Supplementary Figure S1A) revealed the presence of a single population with a hydrodynamic diameter of 390 nm ± 44 nm for PRM-based hPECs and 220 nm ± 17 nm for PEI-based ones, thus confirming the production of monodispersed nanoparticles. Moreover, the polydispersion indexes (PdI) indicated an acceptable degree of monodispersion, being calculated 0.122 and 0.322 for PEI-hPECs and PRM-hPECs, respectively (Supplementary Figure S1A). These values are in line with those reported for monodisperse polymeric and lipid nanoparticles [28]. As already mentioned, the surface charges, expressed as the ζ-potential (Supplementary Figure S1B), indicated the assembly of strong positively charged hPECs. Taken together, these results allowed the following assessment of the efficient delivery of nucleic acids to target cells due to the optimal combination of a relatively small hydrodynamic diameter and the overall positive ζ-potential values of the obtained hPECs.

### 3.2. DNA-Loaded hPEC Analysis and Release

**Figure 2:**
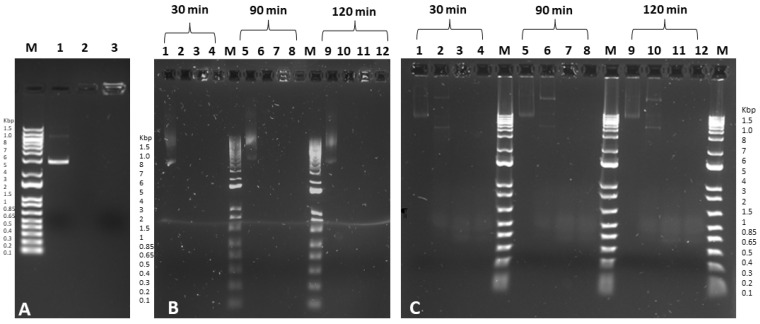
(**A**) Agarose retardation assay of DNA-loaded hPECs. Lane M: DNA marker 1Kb; Lane 1: free DNA; Lane 2: PRM-DNA-loaded hPECs; Lane 3: PEI-DNA-loaded hPECs; (**B**) DNase I protection assay of DNA-loaded hPECs. Lane M: DNA marker 1 Kb; Lanes 1, 5, and 9: free DNA; Lanes 2, 6, and 10 represent free DNA with DNase I; Lanes 3, 7, and 11: DNA-loaded PEI hPECs with DNase I; Lanes 4, 8, and 12: DNA-loaded PRM hPECs with DNase I after incubation with DNase I for 30, 90, and 120 min. DNA refers to EGFP plasmid. (**C**) FBS protection assay of DNA-loaded hPECs. Lane M: DNA marker 1 Kb; Lanes 1, 5, and 9: free DNA; Lanes 2, 6, and 10 represent free DNA with 10% FBS; Lanes 3, 7, and 11: DNA-loaded PEI hPECs with 10% FBS; Lanes 4, 8, and 12: DNA-loaded PRM hPECs with 10% FBS after incubation with FBS for 30, 90, and 120 min. DNA refers to EGFP plasmid. Images are representative of three independent experiments.

The gel retardation assay confirmed the stability and the effective loading of the EGFP pDNA. No visible bands of free DNA were observed for both EGFP-PEI and EGFP-PRM precomplexes as well as for PRM-hPECs and PEI-hPECs (Figure 2A). The efficiency of pDNA encapsulation was then assessed by quantifying the non-loaded free plasmid DNA after the ultracentrifugation of hPECs. A DNA loading efficiency of about 65–70% for both PRM-hPECs and PEI-hPECs was observed, respectively. 

In this frame, DNase I and FBS protection assays were performed. Specifically, the complexation of hPECs with the polycations PRM or PEI at a DNA:polymer ratio of 1:100 was able to protect the loaded pDNA from the action of DNase I and FBS for 30, 90, and 120 min. Partial DNA fragmentation was observed after 2 h only for naked DNA incubated with FBS, while the bands corresponding to loaded hPECs remained in the upper part of the gel (Figure 2B,C). Therefore, it is possible to deduce that the formulated hPECs were able to effectively protect the encapsulated pDNA. It is fair to mention that a good nanoparticle production strategy should couple an effective loading with an essential ability to release the payload upon specific stimuli. The complexation of nucleic acids appeared to be highly efficient in these nanovectors, which is mandatory for ensuring a good protection barrier against the action of nucleases.

The pDNA release was then evaluated under different conditions (Supplementary Figure S2), namely, at pH 7.4, which is the physiological cytosolic value, and at pH 4.5, which is the lysosomal pH. PRM-hPECs released about 50% of the loaded pDNA at pH 7.4 within 24 h, while PEI-hPECs reached a maximum pDNA release of 50% in the first 3 h ( Supplementary Figure S2A,B). The employed polycations were previously proven to be effective in gene delivery for their ability to condense DNA molecules and then to release the cargo in the intracellular environment through the “proton sponge effect” [29,30]. Briefly, in the acidic environment of endosomes, polyamines act as sponges, thus leading to proton accumulation in the lumen. Therefore, chloride ions will diffuse inside to balance the increased membrane potential. As a result, the increased osmotic pressure triggers the swelling of the endosomes and then the release of their content [31,32,33], and, in this case, the plasmid DNA loaded into hPECs. In the proposed nanoformulations, the chitosan of the outer layer of both PEI-hPECs and PRM-hPECs additionally contributed to the proton sponge effect. For this reason, DNA release at pH 4.5 was carefully evaluated to assess the role of acidic pH in disassembling hPECs, thus potentially delivering biologically available pDNA (Supplementary Figure S2A,B). A fast DNA release occurred in PEI-hPECs in the early hours, reaching values approaching 100% release (Supplementary Figure S2A). This suggested that during the endosomal maturation PEI-hPECs undergo disassembling with the potential release of free DNA ready to carry out its biological action. In parallel, the presence of a polyanions as a potential extra stimulus for promoting DNA release from PEI-hPECs was investigated trough the addition of the glycosaminoglycan heparin in the acidic environment. As reported in Supplementary Figure S2C, 100% of DNA release immediately occurred in the first hours, thanks to the combination of the competitive displacement action of heparin together with the acid environment (as already reported in Supplementary Figure S2A). Conversely, the release of DNA from PRM-hPECs at pH 4.5 reached 50% after 24 h (Figure S2B), thus highlighting how the full disassembling of a PRM-based nanoformulation requires an additionally stimulus. Protamine is a very basic nuclear protein composed mainly of the amino acid arginine. The responsiveness of PRM-hPECs in releasing the loaded DNA under the action of proteinase K was thus evaluated (Supplementary Figure S2D). The results indicated a sustained DNA release occurring in the first hours and reaching a percentage around 70% during the observation period of 48 h. This was attributed to the fact that the investigated acid pH was not able to fully disassemble PRM-hPECs, and the external layer of chitosan-TPP may hamper the easy access of the enzyme to the inner core of PRM complexed with DNA. This result highlighted the ability of PRM to act as a substrate for the action of proteases that are often overexpressed in pathological conditions as well as the potential to modulate the responsiveness of PRM to protease over time by properly engineering the external layer based on chitosan-TPP. 

### 3.3. hPEC Protein Corona Analysis

Prior to analyzing the transfection efficiency and the nanovector anti-inflammatory properties, an important key issue for profiling their biological activity was the profiling of the protein adsorbed on the surface of hPECs when suspended in the cell culture medium. Nanoparticles in biological fluids immediately interact with proteins that rapidly bind to their surface, forming a so-called “protein corona” [34,35]. These proteins can confer a new biological identity, which can influence body biodistribution, cellular uptake, and, more importantly, toxicity and inflammation properties [36,37,38]. Before the isolation of the bound proteins by centrifugal steps, DLS analyses of the hPECs suspended in cell culture medium were performed in order to exclude the presence of aggregates. The resulting protein profiles obtained by SDS-PAGE indicated low adsorption of low-molecular-weight proteins by hPECs (Supplementary Figure S3). 

According to the Vroman effect [39], proteins with higher mobility and those more abundant in the serum are the first to interact with the NP surface with soft bonds. These are subsequently replaced by less mobile proteins with greater affinity for the nanoparticle surface that can last longer and are retained, even during endocytosis. This supports the hypothesis of a weak Vroman effect and possibly a weak protein interaction. Notably, both PRM-hPECs and PEI-hPECs produced the same protein corona profile when incubated in a cell culture medium. This indicated that the commonly shared chitosan-TPP outer layer imparted the identified affinity for the proteins present in the cell culture medium. This result was also supported by the analysis of protein coronas of empty hPECs in which the pDNA was substituted by the polyanion heparin during the synthesis procedures (Supplementary Figure S3B). In the same way, the protein profiles resemble those obtained for the DNA-loaded nanoformulations. Hence, the only component responsible for the nanoparticle interactions with proteins is the chitosan-TPP added in the final step of synthesis. The main protein band showed by hPECs (loaded and empty, Supplementary Figure S3A,B) locates similarly to the main band of cell culture medium (lane 4, Supplementary Figure S3A, lanes 4–5, Supplementary Figure S3B) that corresponds to BSA, the most abundant FBS protein employed to supplement the medium. All the hPECs investigated here retained this main band over times from 1 h of incubation to 24 h, further demonstrating weak and unspecific interactions between the proteins and the nanostructure. The evidence of weak and unspecific protein interactions, along with the sizes and the cationic nature of hPECs, suggested the possibility of efficient delivery of the encapsulated pDNA while minimizing interactions with the biomacromolecules present in the biological fluids.

### 3.4. Uptake and In Vitro Transfections

**Figure 3:**
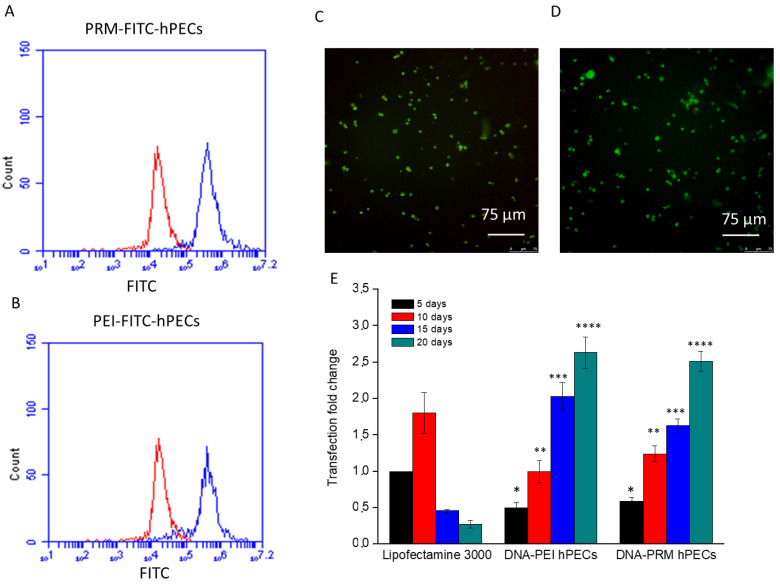
Cytofluorimetry analysis of FITC-PRM-hPEC ((**A**), blue line) and FITC-PEI-hPEC ((**B**), blue line) uptake by THP-1 cells and relative CLSM merge images of FITC-PRM-hPECs (**C**) and FITC-PEI-hPECs (**D**) after 24 h of treatment. Non-treated cells (CTR) were used as control (red line in (**A**,**B**)). Scale bars: 75 μm. Cytofluorimetry analysis (**E**) of treated THP-1 cells in order to evaluate the transfection, expressed as fold change over time. Lipofectamine 3000 transfection was assigned an arbitrary value of 1, and the transfection efficacy of hPEC formulations was normalized to the efficiency of lipofectamine 3000 (absolute value of transfection of Lipofectamine 3000 was 15.5%). DNA refers to EGFP plasmid. Representative measurements of three distinct sets of data. *, **, ***, **** indicate *p*-values < 0.05 for Student’s *t*-test.

Quantitative uptake was assessed by cytofluorimetry (Figure 3A,B and Supplementary Figure S4) after 24 h of incubations with FITC-labeled hPECs. The internalization efficacy was about 88% for PRM-hPECs and 83% for PEI-hPECs. A robust green fluorescence related to the hPECs uptake was also reported within the cytoplasm after 24 h of incubation with fluorescent FITC-labeled PRM or PEI hPECs (Figure 3C,D).

To analyze how stable the transfection induced by hPECs was over time, plasmids carrying the EGFP gene were loaded into PEI- and PRM-hPECs, and the transfection was conducted on a macrophage precursor cell line (THP-1). During all the experiments, the cell culture medium was never completely replaced. Instead, additions to the medium were made in order to avoid hPEC removal. The cytofluorimetry results (Figure 3E) indicate that the transfection efficacy, expressed as fold change using Lipofectamine 3000 as a reference standard, of THP-1 cells by hPECs showed a lower efficacy when compared to Lipofectamine 3000 in the initial stage. However, this lack of efficiency was related only to the first days of exposure. Conversely, the percentage of GFP-positive cells was higher in hPEC-treated samples when compared to the Lipofectamine-treated samples after 10–20 days of THP-1 culture in the presence of PEI- and PRM-hPECs. These behaviors were related to the fact that after cellular uptake Lipofectamine 3000 fused with the cytoplasmic membrane and released all its contents into the cytoplasm, obtaining a burst in the first hours of transfection, which decreased in time. On the other hand, our nanovectors degraded slowly, and in this way a sustained release of their cargo over time was possible, obtaining transfection efficiencies, even at much longer intervals. These data revealed the abilities of these nanocomplexes to perform an efficient and sustained release of the genetic cargo over time (up 20 days).

### 3.5. Analysis of Cytotoxicity and Inflammatory Response

**Figure 4:**
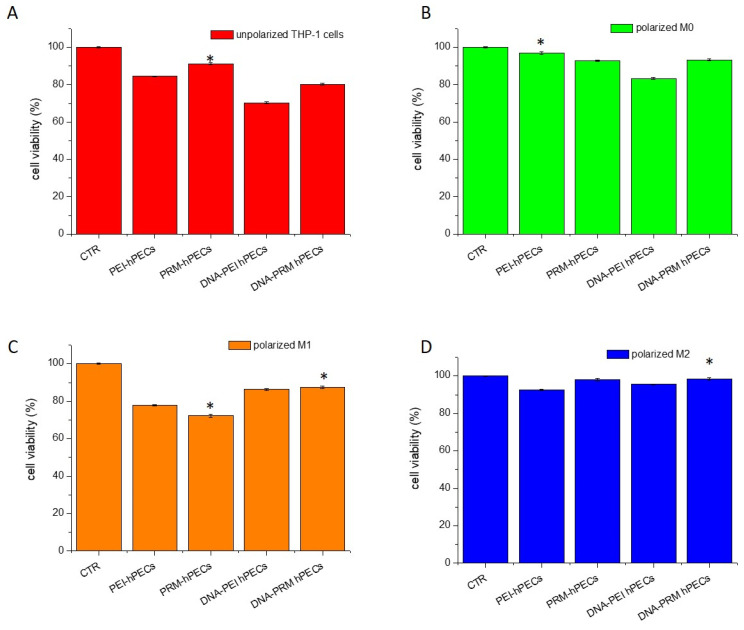
MTT assay on THP-1 cells after 24 h of incubation with different hPEC formulations without polarization (**A**), after M0 polarization (**B**), after M1 polarization (**C**), and after M2 polarization (**D**). Representative measurements of three distinct sets of data. * indicates *p*-values of <0.05 for Student’s *t*-test.

Macrophages can mediate pro-inflammatory or anti-inflammatory processes. M1 macrophages release high levels of cytokines, reactive oxygen species (ROS), inducible nitric oxide synthase (INOS) with the production of nitric oxide (NO), and cyclooxygenase (COX)-2 to fight foreign antigens. The above activated pathways led to an increase in the recruitment of immune cells and the engulfment and neutralization of external antigens, giving those cells an antigen-presenting role [40,41]. Meanwhile, M2 macrophages try to resolve infections through the release of anti-inflammatory signals, showing an anti-inflammatory activity in inflammatory diseases [40,42]. They have also been shown to develop protumor characteristics and promote tumor growth and metastasis [42]. From this point of view, an in-depth investigation of the biological effects exerted by the obtained hPECs on macrophages is of paramount importance to further elucidate the mechanisms of action of nanomaterials for therapeutic application in living cells and, importantly, to ensure a non-inflammatory framework so that they can safely perform their action. The in vitro cell toxicity and inflammation responses of the hPEC formulations were tested by MTT, the live/dead assay, and ROS and NO production assessment. The evaluation the of activity inhibition of superoxide dismutase (SOD) was also evaluated on polarized THP-1 cells after 48 h of incubation. THP-1 cells were treated separately to induce the three stages of differentiation (M0-M1-M2), as indicated in the Materials and Methods Section. The polarization status of the cells (M0-M1-M2) was assessed by morphological analyses after treatments with different cytokines (see Supplementary Figure S5). The viability was significantly higher with the different DNA-loaded hPECs (range 90–98%, Figure 4). 

**Figure 5:**
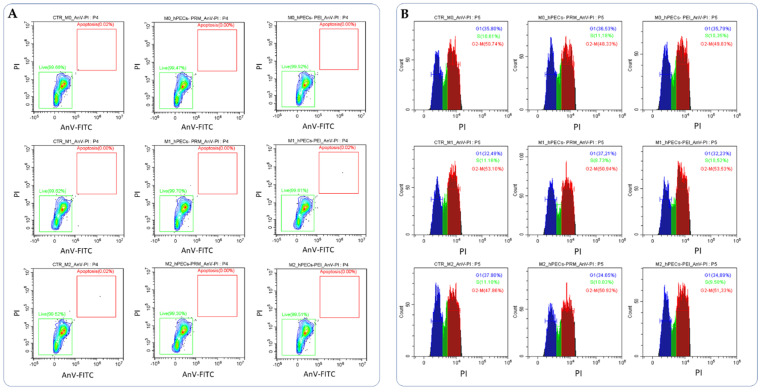
Cytofluorimetric analysis of cell apoptosis (**A**) and cell cycle (**B**) of THP-1 cells after 24 h of incubation with different hPEC formulations after M0, M1, and M2 polarization after 24 h of treatments compared with untreated control cells (CTR). Representative images of three independent experiments.

The metabolic cytotoxicity analysis showed that cell viability was not affected by the treatment with hPECs. These results were confirmed by the live/dead assays (Figure 5A) and cell cycle analyses (Figure 5B).

In addition, we evaluated the production of ROS and NO, which determine cell death, and the inhibition of SOD. The investigations did not reveal any increase in ROS or NO production (Supplementary Figures S6 and S7) in cells treated with the hPEC formulations. These results were confirmed by the analysis of the activity of superoxide dismutase (SOD) that did not show inhibition in the cells incubated with both hPECs (Figure S8). 

Another important issue to address in the formulation of non-viral delivery systems is the possible induction of inflammation. One of the most important players in inflammation are macrophages. Thanks to the expression and activation of multiple surface receptors able to recognize non-autologous patterns [43,44], NF-κB is quickly activated and induces the transcription of several pro-inflammatory cytokines, chemokines, and inflammatory mediators [45,46,47]. 

**Figure 6:**
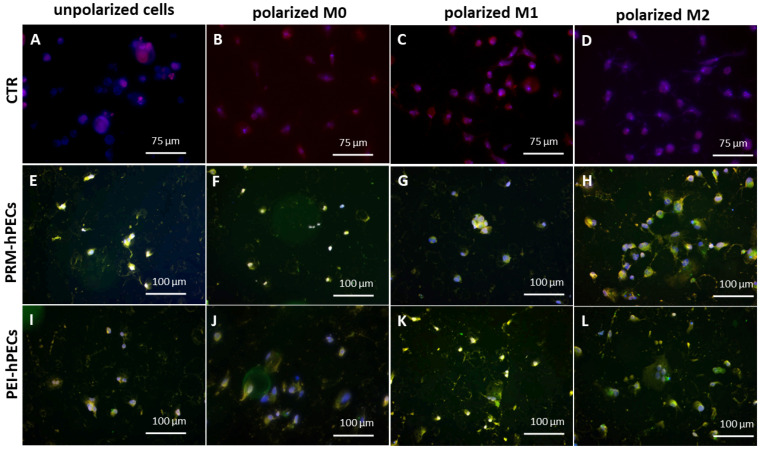
Fluorescence merge images of unpolarized THP-1 cells and polarized M0, M1, and M2 THP-1 cells treated for 48 h with FITC-PRM (**E**–**H**) or FITC-PEI hPEC (**I**–**L**). Non-treated cells were used as control ((**A**–**D**) CTR). In red, NF-κB factors; in green, hPECs; and in blue, nuclei. Scale bars: 75 μm and 100 μm. Representative images of three independent experiments are shown.

To investigate whether the hPECs were safe to use and did not induce inflammation activation, a deeper analysis on the inflammation pathway using fluorescent microscopy and cytofluorimetry analysis was conducted on all the three stages of the differentiation of THP-1. The fluorescence microscopy analysis of NF-κB expression and distribution revealed that hPEC treatments did not lead to the activation of the inflammation pathway in the three polarization stages (Figure 6). These data were confirmed by the evaluated expression of NF-κB by cytofluorimetry analysis (Figure S9).

Furthermore, along with the non-inflammatory properties, the hemocompatibility of the proposed formulations was assessed in order to provide a crucial parameter for their potential to be safely administered in vivo. The results reported in Supplementary Figure S10 showed that the hemocompatibility of our formulations was significantly higher (about 2% hemolysis) for both PRM-hPECs and PEI-hPECs when compared to Lipofectamine 3000 (about 15% hemolysis). Taken together, these results further support the strategic design of the proposed hPECs that allow the formulations to be biocompatible, thanks to the external layer of chitosan, while performing efficient gene delivery. 

## 4. Conclusions

In this study, hPECs with a multicomponent structure for non-viral gene delivery were developed. The positive charge of polycations, such as linear polyethylenimine (PEI) and protamine sulfate (PRM), was exploited for encapsulating the negatively charged pDNA in the inner core. This provided the right balance between the protection of the genetic cargo from nucleases and sustained delivery over time under particular stimuli. The outer layer composed of chitosan imparted to both hPEC formulations weak interaction capabilities with the protein present in the cell culture medium. The combination of these features was responsible of the high transfection efficiency over time as well as their biocompatibility and anti-inflammatory properties, thus paving the way to a safe employment of nanoparticle-based solutions for gene therapy in several challenging conditions. Fort this reason, the outcomes for the formulated hPECs represent a step forward towards the exploitation of nanotechnology-based solutions in cancer immunotherapy, especially in CAR-T production, thus overcoming the drawbacks derived from viral vectors. 

## Data Availability

The data presented in this study are available in article.

## Supplementary Materials

The following supporting information can be downloaded at: https://www.mdpi.com/article/10.3390/pharmaceutics14071310/s1, Figure S1: Reported DLS and ζ-potential analyses of nanoformulations complexed with linear polyethyleneimine or protamine (PEI or PRM) stabilized with lecithin as well as size distribution analyses of hPECs imaged by AFM; Figure S2: Reported DNA release from hPECs under different stimuli for a time window of 48 h; Figure S3: Reported SDS-PAGE of protein corona obtained from EGFP-loaded PRM-hPECs (lanes 1, 2, and 3) and EGFP-loaded PEI-hPECs (lanes 5, 6, and 7) after incubation with RPMI at 37 °C for 1 h (lanes 1 and 5), 6 h (lanes 2 and 6) and 24 h (lanes 3 and 7). SDS-PAGE of protein corona obtained from empty PRM-hPECs (lanes 1, 2, and 3) and PEI-hPECs (lanes 6, 7, and 8) after incubation with RPMI at 37 °C for 1 h (lanes 1 and 6), 6 h (lanes 2 and 7) and 24 h (lanes 3 and 8); Figure S4: Reported dot plots of cytofluorimetry analysis of CTR and uptake by THP-1 cells of FITC-PRM-hPEC and FITC-PEI-hPECs; Figure S5: Reported polarization status of the cells (M0, M1, and M2); Figure S6: Reported cytofluorimetric analysis of production of ROS on polarized THP-1 cells (M0, M1, and M2) after 24 h of incubation with different hPEC formulations; Figure S7: Reported cytofluorimetric analysis of NO production on polarized THP-1 cells (M0, M1, and M2) after 24 h of incubation with different hPEC formulations; Figure S8: Reported cytofluorimetric analysis of SOD activation inhibition on polarized THP-1 cells (M0, M1, and M2) after 24 h of incubation with different hPEC formulations; Figure S9: Reported cytofluorimetry analysis of NF-κB expression in unpolarized THP-1 cells and polarized M0, M1, and M2 THP-1 cells treated for 48 h with PRM-hPECs and PEI-hPECs; Figure S10: Reported hemolytic assay carried out after 1 h of incubation at 37 °C with DNA-Lipofectamine 3000, empty PEI, or PRM hPECs and DNA-PEI or PRM hPECs.

## Abbreviations

hPECs, hybrid polyelectrolyte nanocomplexes; pDNA, plasmidic DNA; PEI, polyethyleneimmine; PRM, protamine; TPP, tripolyphosphate; CAR, chimeric antigen receptor; APC, antigen-presenting cells; CH, chitosan; EGFP, green fluorescence protein; MMW, medium-molecular-weight; PVA, poly(vinyl alcohol); FITC, fluorescein isothiocyanate; ζ, zeta potential; DLS, dynamic light scattering; FBS, fetal bovine serum; BME, 2-mercaptoethanol; PMA, phorbol 12-myristate 13-acetate; LPS, lipopolysaccharides; IFN-γ, interferon-gamma; IL-4, interleukin-4; IL-13, interleukin-13; ROS, reactive oxygen species; INOS, nitric oxide synthase; NO, nitric oxide; SOD, superoxide dismutase; THP-1, Human monocytic cell line.
