# Supplementary material for: Hybrid Polyelectrolyte Nanocomplexes for Non-Viral Gene Delivery with Favorable Efficacy and Safety Profile
Source: Pharmaceutics. 2022 Jun 21;14(7):1310. doi: 10.3390/pharmaceutics14071310 (PMC9323431; doi:10.3390/pharmaceutics14071310)
Supplement: Supplementary file 1 [file pharmaceutics-14-01310-s001.zip › pharmaceutics-1761032-supplementary.pdf]

Supplementary Information

for

Hybrid polyelectrolyte nanocomplexes for non-viral gene delivery with favorable efficacy and safety profile

Gabriele Maiorano[1&], Clara Guido[1,2&], Annamaria Russo[1], Andrea Giglio[1], Loris Rizzello[3,4], Mariangela Testini[1], Barbara Cortese[5], Stefania D'Amone[1], Giuseppe Gigli[1,2] and Ilaria E. Palamà[1*]

[1] Nanotechnology Institute, CNR-NANOTEC, Monteroni street - 73100 Lecce, Italy; gabriele.maiorano@nanotec.cnr.it (G.M); clara.guido@nanotec.cnr.it (C.G); annamaria.russo@nanotec.cnr.it (A.R); andrea.giglio@nanotec.cnr.it (A.G.); mariangela.testini@nanotec.cnr.it (M.T.); stefania.damone@nanotec.cnr.it (S.DA.); ilaria.palama@nanotec.cnr.it (I.E.P.)
[2] Dep. of Mathematics and Physics, University of Salento, Monteroni street - 73100 Lecce, Italy; giuseppe.gigli@unisalento.it (G.G.)
[3] University of Milan, Department of Pharmaceutical Sciences (DISFARM), G. Balzaretti 9 street - 20133 Milan, Italy; loris.rizzello@unimi.it (L.R.)
[4]National Institute of Molecular Genetics (INGM), Francesco Sforza 35 street - 20122 Milan, Italy
[5]Nanotechnology Institute, CNR-NANOTEC, c/o La Sapienza University, P.le A. Moro- 00185 Rome, Italy; barbara.cortese@nanotec.cnr.it (B.C.)

[&] Contributed equally

* Correspondence: ilaria.palama@nanotec.cnr.it

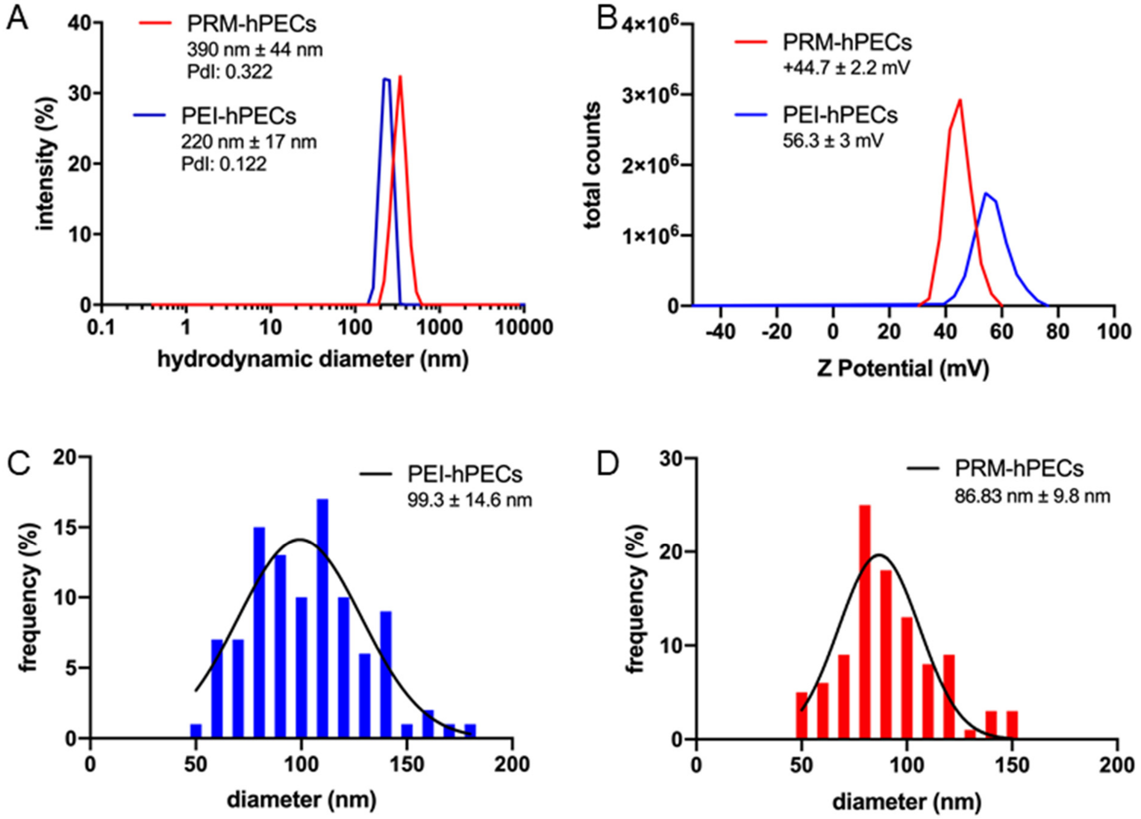

Figure S1. (A) DLS analyses of hPECs after dialysis and resuspension in PBS, pH 7.4. Hydrodynamic diameter and polydispersion indexes are reported. (B) ζ-potential analyses of the assembled hPECs. (C,D) Size distribution analyses with Gaussian fits of PEI hPECs and PRM-hPECs, respectively, obtained by measuring of at least 100 particles from each AFM image (reported in Figure 1C,D).

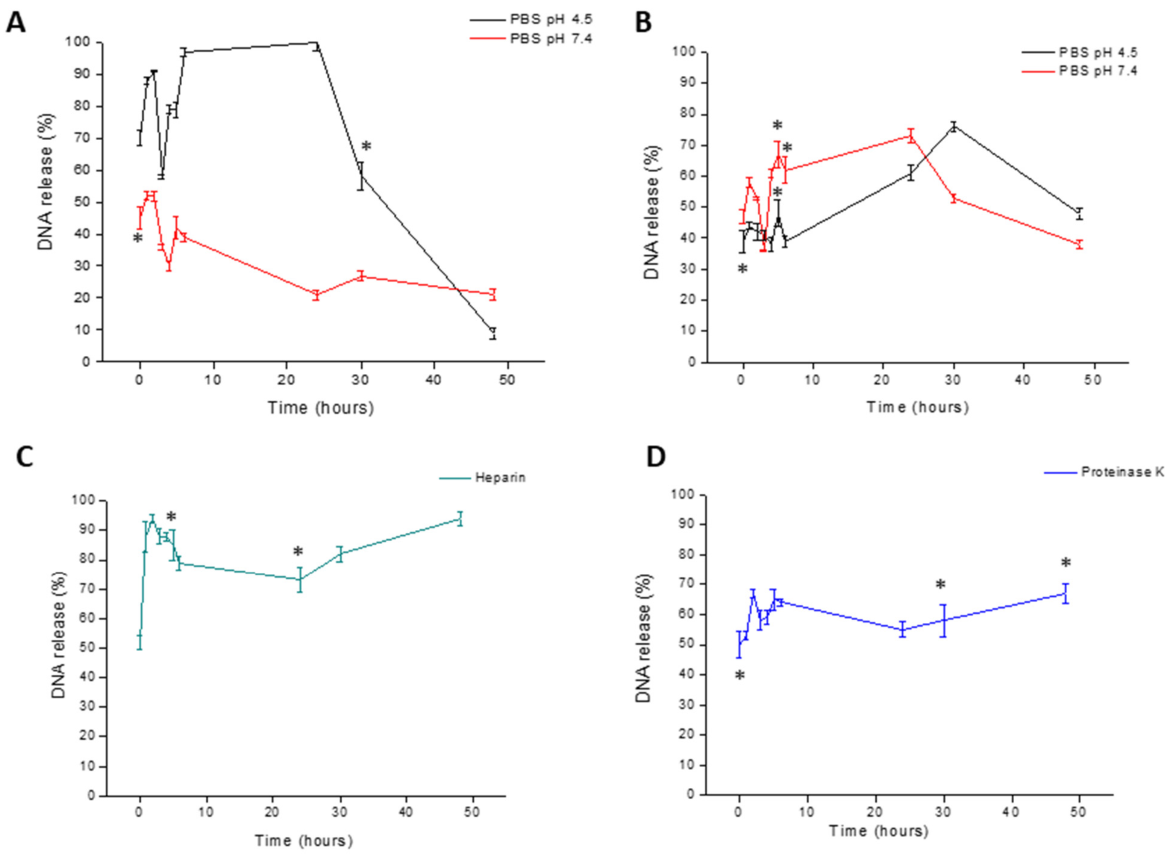

Figure S2: DNA release from hPECs under different stimuli for a time window of 48 hours. A) DNA-loaded PRM hPECs incubated at pH 4.5 and 7.4. B) DNA-loaded PEI hPECs incubated at pH 4.5 and pH 7.4. C) DNA-loaded PRM hPECs incubated with proteinase K. D) DNA-loaded PEI hPECs incubated heparin. DNA refers to EGFP plasmid. Representative measurements of three distinct sets of data * indicates P-values of <0.05 for t-Student test.

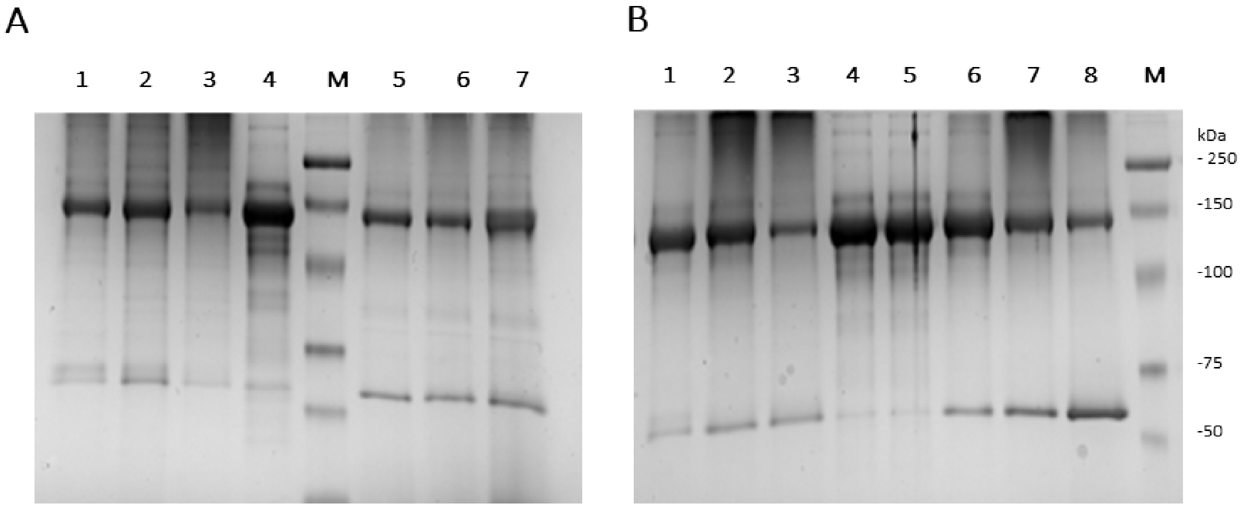

Figure S3. In panel A, SDS-PAGE of protein corona obtained from EGFP loaded PRM-hPECs (lanes 1,2,3) and EGFP loaded PEI-hPECs (lanes 5,6,7) after incubation with RPMI at 37 °C for 1 hour (lanes 1,5), 6 hours (lanes 2,6) and 24 hours (lanes 3,7). Lanes 4: only RPMI medium. Lane M: protein marker. In panel B, SDS-PAGE of protein corona obtained from empty PRM-hPECs (lanes 1,2,3) and PEI-hPECs (lanes 6,7,8) after incubation with RPMI at 37 °C for 1 hour (lanes 1,6), 6 hours (lanes 2,7) and 24 hours (lanes 3,8). Lanes 4 and 5: only RPMI medium. Representative images of three independent experiments.

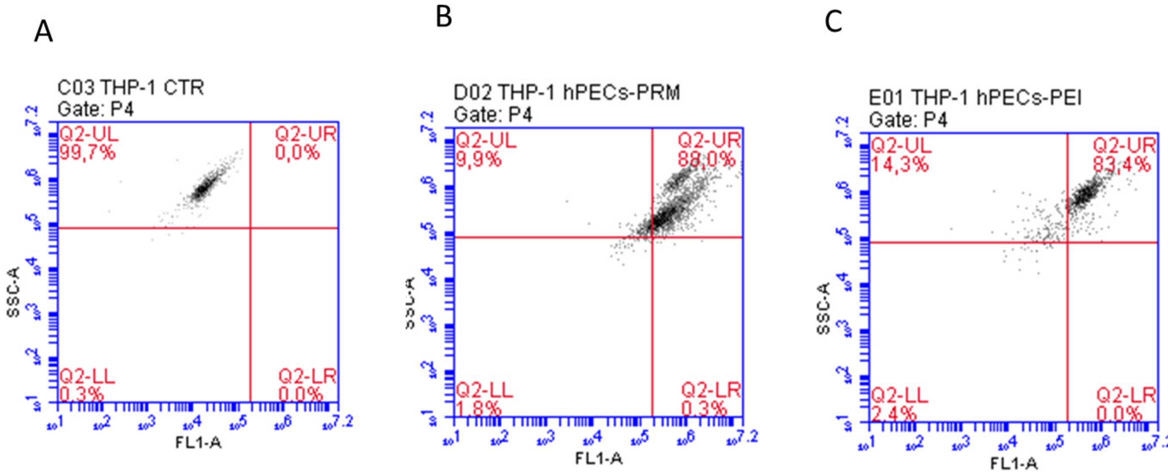

Figure S4. Dot plots of cytofluorimetry analysis of CTR (A), and uptake by THP-1 cells of FITC-PRM-hPEC (B) and FITC-PEI-hPECs (C).

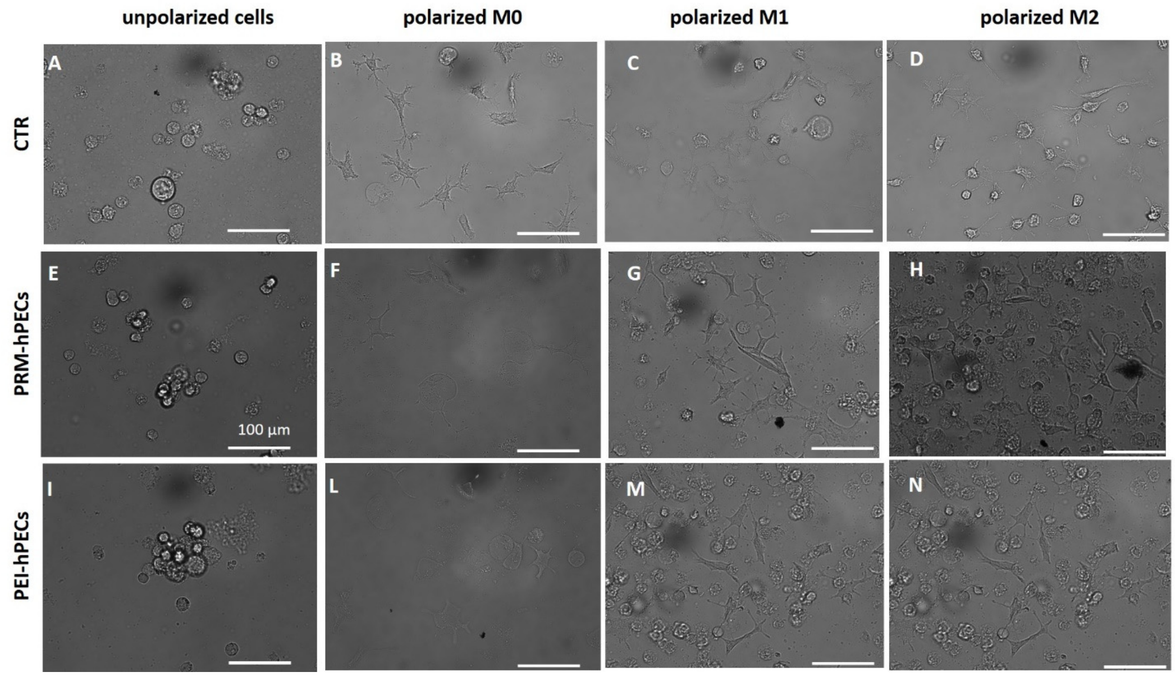

Figure S5. Polarization status of the cells (M0- M1 - M2) was assessed by morphological analyses under the microscope after treatment with different cytokines. Scale bars: 75 µm

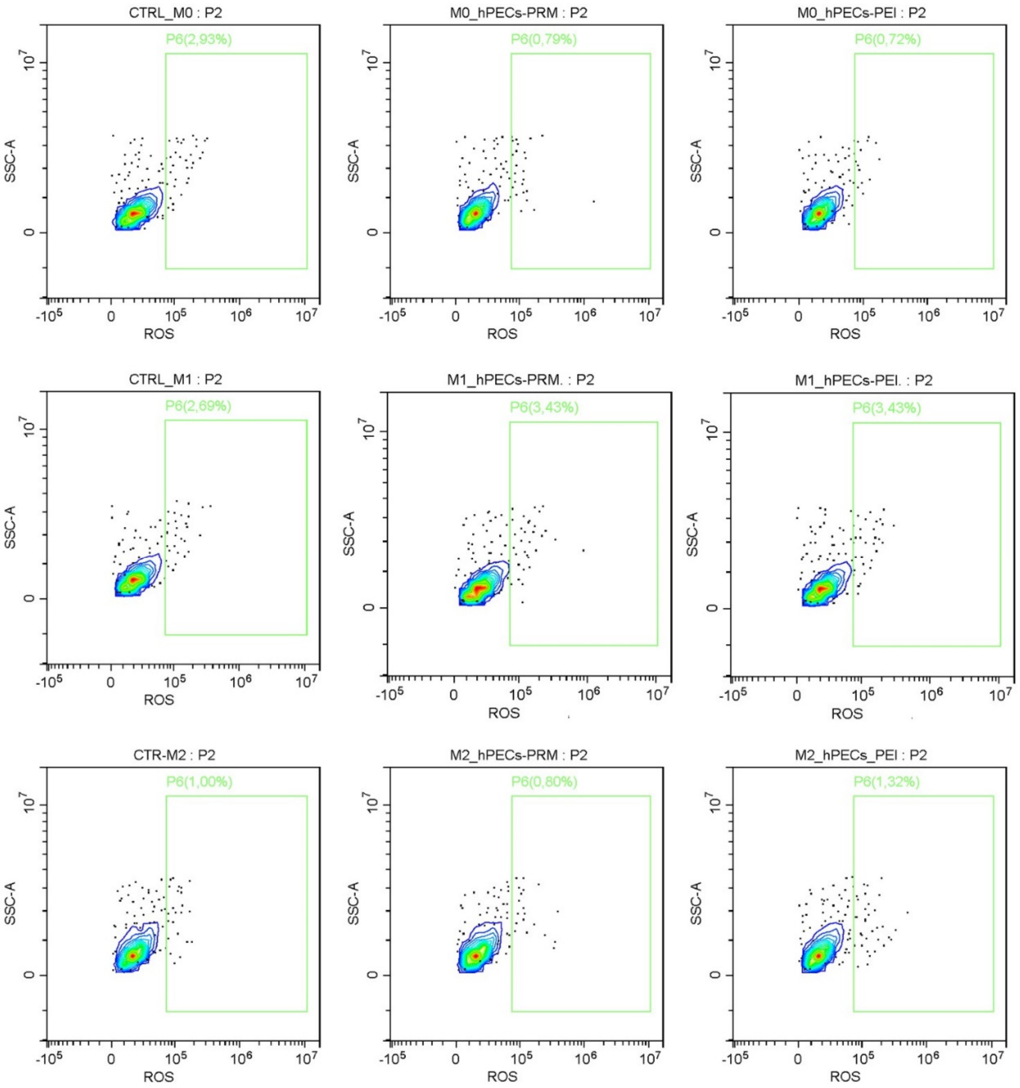

Figure S6. Cytofluorimetric analysis of production of ROS on polarized THP-1 cells (M0, M1, M2) after 24 hours of incubation with different hPECs formulations compared with untreated control cells (CTR).

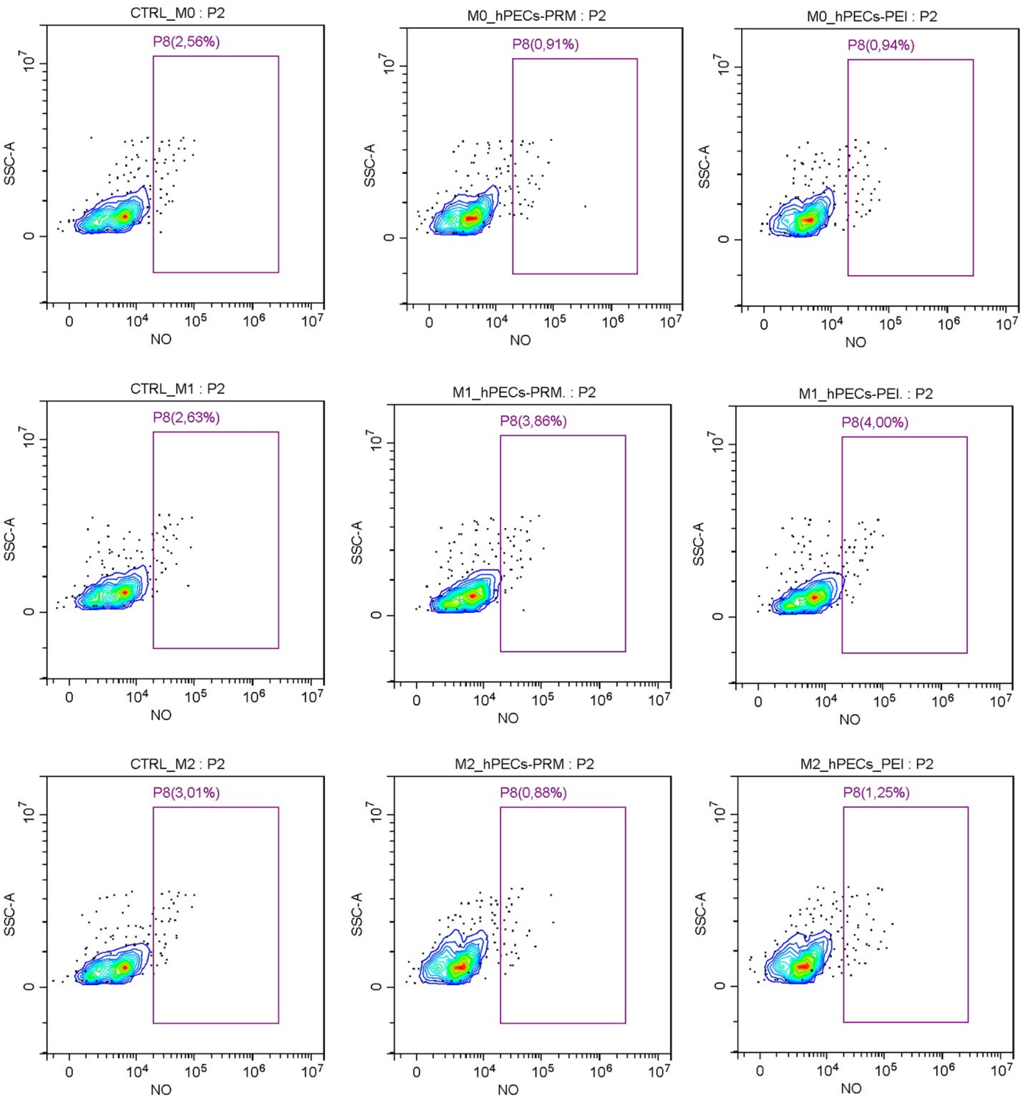

Figure S7. Cytofluorimetric analysis of NO production on polarized THP-1 cells (M0, M1, M2) after 24 hours of incubation with different hPECs formulations compared with untreated control cells (CTR).

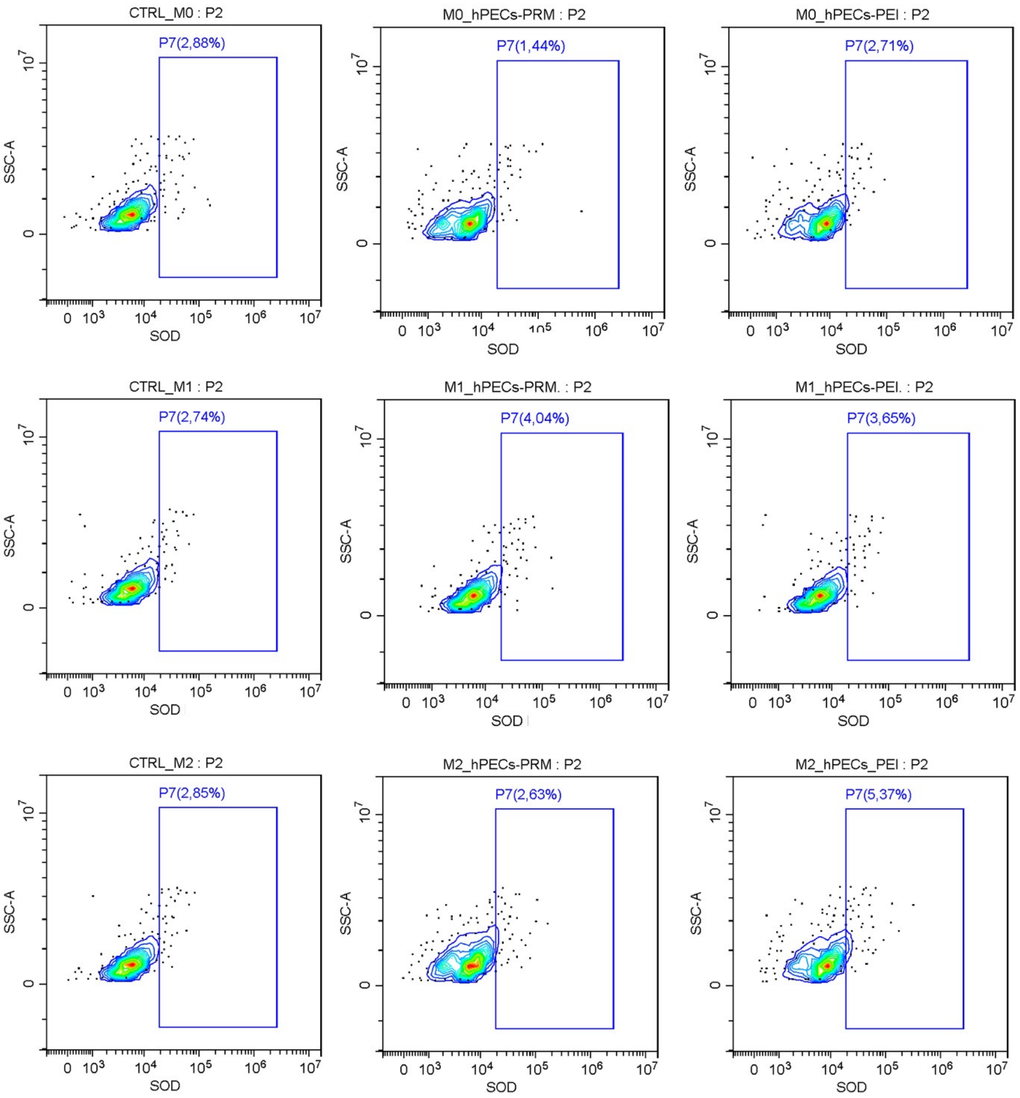

Figure S8. Cytofluorimetric analysis of SOD activation inhibition on polarized THP-1 cells (M0, M1, M2) after 24 hours of incubation with different hPECs formulations compared with untreated control cells (CTR).

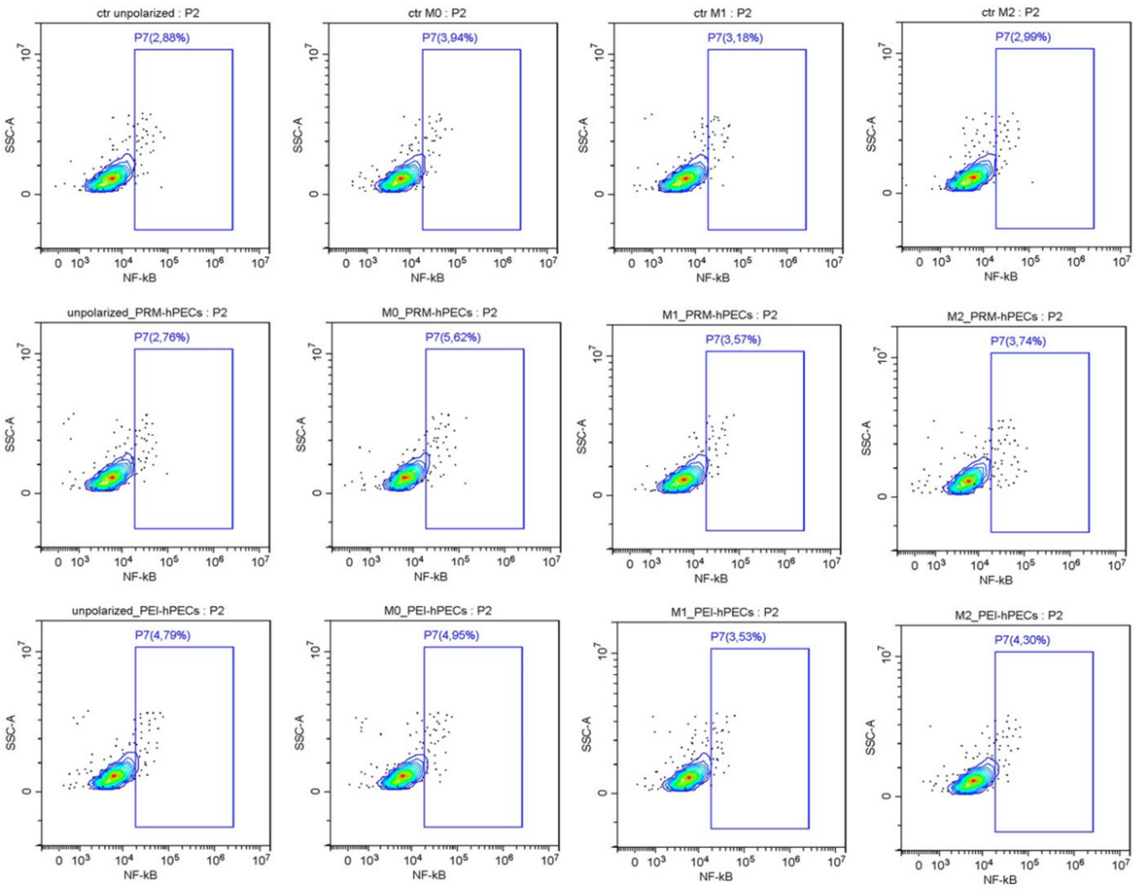

Figure S9. Cytofluorimetry analysis of NF-κB expression in unpolarized THP-1 cells and polarized M0, M1, M2 THP-1 cells treated for 48 hours with PRM-hPECs and PEI-hPECs.

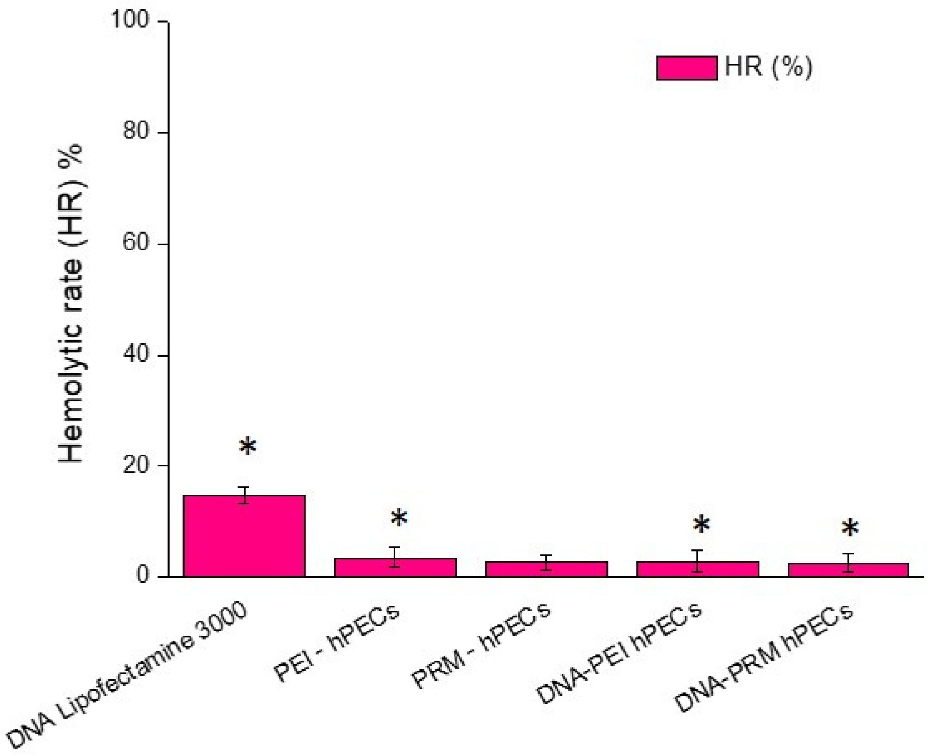

Figure S10. Hemolytic assay carried out after 1 hour of incubation at 37°C with DNA-Lipofectamine 3000, empty PEI or PRM hPECs and DNA-PEI, or-PRM hPECs. DNA refers to EGFP plasmid. Representative measurements of three distinct sets of data * Indicates P-values of <0.05 for t-Student test.
